# Coproduction of a resource sharing public views of health inequalities: An example of inclusive public and patient involvement and engagement

**DOI:** 10.1111/hex.13860

**Published:** 2023-09-13

**Authors:** Charlotte Parbery‐Clark, Rosemary Nicholls, Lorraine McSweeney, Sarah Sowden, Joanne Lally

**Affiliations:** ^1^ Population Health Sciences Institute, Faculty of Medical Sciences Newcastle University Newcastle upon Tyne UK; ^2^ Patient and Public Involvement Representative and Consumer Panel Member NIHR Research Design Service (RDS) North East North Cumbria (NENC) Newcastle upon Tyne UK

**Keywords:** co‐production, health inequalities, PPIE

## Abstract

**Background:**

UNderstanding Factors that explain Avoidable hospital admission Inequalities—Research study (UNFAIR) addresses how to reduce health inequalities, particularly for avoidable hospital admissions. Our Patient and Public Involvement and Engagement (PPIE) members broached that health inequalities are complex, challenging to understand and communicate. They identified a need to explore diverse views, including people who have a higher risk of health inequalities. With limited public‐facing resources relating to the public's understanding or emotions around health inequalities, this project aimed to fill this gap using co‐leadership and co‐production.

**Methods:**

Members of the public worked with researchers to co‐produce and run PPIE workshops. This project was co‐led by a member of the public and a researcher. One online workshop open to anyone in England accompanied by three face‐to‐face workshops were held. Public contributors, including people living in diverse communities, were invited. Inclusive involvement opportunities were offered including flexible ways of involvement and remuneration. To strengthen the key messages' rigour, transcriptions of the audio‐recordings from each workshop, with facilitator notes, were analysed using thematic analysis. From the key messages, an animation was co‐produced with public contributors with the public's voice being integral throughout.

**Key Messages:**

A total of 58 people took part capturing intersecting and multiple dimensions of marginalisation including people with a range of ages, genders, ethnicities, socioeconomic backgrounds, and members of communities who face exclusion (including people with learning difficulties and experiencing ill‐health). The animation highlighted powerful lived experience, for example, some people are dying earlier than expected. Health inequalities conjured up powerful emotions, such as anger and hopelessness. Public views of how to address health inequalities included respecting, accepting and valuing everyone, regardless of, for example, where people live. The animation is publicly available for use by anyone, including decision makers across the health and care system.

**Conclusions:**

Through co‐leadership and co‐production, this project is an example of inclusive PPIE. This project provided a way for the public's voice to influence policy and practice to inform understanding and action to address health inequalities. The animation provides powerful insights into what health inequalities mean to people with examples of lived experience and corroborates the moral argument for action by decision makers.

**Patient and Public Contribution:**

Members of the public, including people who were affected or at higher risk of health inequalities, co‐led this project and were involved as co‐creators and developers from the inception of the project to completion. Their involvement was integral and documented in full throughout the project.

## 1 INTRODUCTION

Health inequalities are differences in health between different groups of people and are considered to be unfair and avoidable.^1^ The UNderstanding Factors that explain Avoidable hospital admission Inequalities—Research study (UNFAIR) Patient and Public Involvement and Engagement (PPIE) members raised that health inequalities are complex and can be difficult to both understand and communicate. Being able to communicate complex concepts like health inequalities is the first step in increasing the involvement of members of the public in the research cycle, as public contributors, or as research participants. Despite several public‐facing resources on the factors that relate to our health,^2^, ^3^, ^4^ there are limited public‐facing resources describing the public's understanding and emotions around health inequalities, therefore this project was proposed. This project was not research designed to create new knowledge; it was designed to involve members of the public in creating a resource to help explain what health inequalities mean to members of the public. This resource could then be used in several ways including researchers using it to help explain, as well as explore understanding of this concept at the start of the research cycle, and describe the research proposed to examine health inequalities. Additionally, knowing how the public view and understand health and health inequalities is important to help frame the conversation and communicate the evidence in the most effective way to the required audience.^5^ For example, to aid the UK government's proposed levelling‐up agenda, a programme of work aimed to help reduce geographical inequalities, researchers have made policy recommendations which included ‘to broaden the public narrative on health outcome disparities from being perceived as a predominantly health service issue (dealing with the impact) to a social/structural issue that everyone needs to invest in’.^6^ ^(p. 4)^ It was suggested that a public conversation could assist this discussion.^6^

Members of the public were integral to the design of this project, including co‐producing and co‐delivery of the workshops, developing the resource as well as disseminating the key messages. The National Institute for Health and Care Research's (NIHR) approach to public involvement and coproduction underpinned this project whereby members of the public and researchers were active partners with shared power and responsibility from inception to completion of the project.^7^ Establishing and maintaining relationships as well as the inclusion of all perspectives with mutual respect were core components.^7^ The project's aims were to explore public understanding and views of health inequalities by involving a diverse group of people and to develop a public‐facing resource describing how members of the public view health inequalities. Three objectives were identified:

1. understand what health inequalities mean to people,
2. explore how members of the public feel about health inequalities,
3. explore people's views about what can be changed to address health inequalities.

Furthermore, to address the lack of examples of PPIE involving members of the public in the exploration of more complex/abstract concepts, such as health inequalities and public health, this project aimed to provide an example of an inclusive PPIE journey for other researchers undertaking related projects.

## 2 METHODS

### 2.1 Theoretical considerations

The work was theoretically underpinned by the 3A^3^ Framework of Participation. This framework brings together theory and concepts to provide new insights into how the key elements that make up public participation, including instances of co‐production and co‐creation,^8^ interact. This framework considers who we hoped to involve including how they would be recruited and what their roles would be, where/how involvement would occur as well as the aims, rationale and outcomes from involvement and potential issues that may arise.^8^ Furthermore, when designing, implementing and evaluating the workshops, we followed UK Standards for Public Involvement^9^ and Table 1 summarises the project's activities according to these standards. The GRIPP2 reporting checklist^10^ is available in the Supporting Information.

**Table 1:** Summary of how the six domains of the UK standards for public involvement^9^ were incorporated into our PPIE project plan.

UK standards for public involvement	Summary of standard	Summary of project's activities
Inclusive opportunities	‘Offer public involvement opportunities that are accessible and that reach people and groups according to research needs’.	Idea came from the Core PPIE Advisory Team (4 UNFAIR PPIE members) who encouraged researchers to seek more diverse views including people who are affected or at higher risk of health inequalities.
		The Core PPIE Advisory Team and UNFAIR research team after considering the pros and cons decided not to develop inclusion/exclusion criteria for the workshops to be as inclusive as possible.
		Reached out to local and national networks who work with diverse communities to promote the workshops.
		Provided choices about how to express an interest in attending the workshops.
		Public contributors' preferred choice for communication was prioritised, where possible.
		Offered workshops both online and in‐person locally.
		Remuneration was offered in line with NIHR guidance with prompt submission to Newcastle University's finance team post involvement to reduce delays.
Working together	‘Work together in a way that values all contributions, and that builds and sustains mutually respectful and productive relationships’.	From the project's start, the Core PPIE Advisory Team and the lead author agreed on how they would work together, which included adding extra progress updates about the UNFAIR research programme.
		The aims, objectives, structure (including which images to use) of the workshops were co‐produced with the Core PPIE Advisory Team and a community group.
		All feedback was welcomed and any feedback, including any disagreements, was seen as opportunities for growth.
		It was difficult when feedback conflicted or was not feasible, so approaches (e.g., voting on options) were developed to overcome these challenges.
Support and learning	‘Offer and promote support and learning opportunities that build confidence and skills for public involvement in research’.	Support of the lead and last author was always available. This included by telephone and/or email.
		Opportunities for the Core PPIE Advisory Team were offered to co‐chair sessions with staff members, which included supportive planning sessions.
		Gathered feedback in the evaluation questionnaire with incorporation into future sessions.
		Meetings with the Core PPIE Advisory Team included sharing our learning together.
Communications	‘Use plain language for well‐timed and relevant communications, as part of involvement plans and activities’.	The Core PPIE Advisory Team reviewed and provided feedback on the public‐facing information.
		Flexibility in communications, which included promotion of the workshops (e.g., by flyer or email) as well as how to sign up for workshops, was provided.
		A communication plan for each session and a dissemination plan were created and followed.
		Gathered feedback at each session, via the evaluation questionnaire.
		Deadlines needed to be pushed back at times due to how time consuming some of the steps were.
Impact	‘Seek improvement by identifying and sharing the difference that public involvement makes to research’.	The co‐leads and the Core PPIE Advisory Team reflected on the experiences and learning throughout the project.
		Gathered feedback via the evaluation questionnaire after each session.
		Monitored activity on social media during the animation's launch as well as ongoing monitoring of the number of views on YouTube.
		Additional spin‐off projects are in place to share the learning (one blog and additional animations).
Governance	‘Involve the public in research management, regulation, leadership and decision making’.	Co‐leadership between the researchers and members of the public was agreed upon.
		The public voices were an integral part of the decision making and involved in all key decisions.
		Any personal information was protected in line with governance principles including sharing with public contributors why, how and for how long any personal information would be stored.

Abbreviations: NIHR, National Institute for Health and Care Research; PPIE, Patient and Public Involvement and Engagement; UNFAIR, UNderstanding Factors that explain Avoidable hospital admission Inequalities—Research study.

### 2.2 Development of the core PPIE advisory team

Four members of the public have been involved in the research design of UNFAIR since its inception. They first became involved in the research project when the principal investigator of UNFAIR presented the research proposal to gather PPIE feedback at a NIHR Research Design Service (RDS) Public Involvement Consumer Panel. This group of four members of the public formed the Core PPIE Advisory Team with one member being a co‐applicant and co‐lead (R. N.) on this particular project. Regular meetings with the Core PPIE Advisory Team and lead author (C. P‐C.) were held to co‐produce the project. This included agreeing roles, responsibilities and how we would work together, including the creation of our terms of reference (TOR).^11^, ^12^, ^13^ For example, our TOR helped create a sharing of power including setting out that decisions would be made by consensus and included which steps to take if this was not possible. After each Core PPIE Advisory Team meeting, a summary of the key discussion points and the key decisions that were made was shared with all members via email. Through these meetings and an additional meeting with a community group, we co‐designed the content and delivery of the workshops, which are discussed in more detail in Section 2.3. with information about the community groups we worked with.

### 2.3 Overview of the workshops

To understand how the public views health inequalities and their ideas of what could be changed, we organised both online and in‐person workshops with members of the public. One online workshop open to anyone in England and then two local North East of England face‐to‐face events with community groups (following COVID‐19 restrictions at the time) were undertaken in the summer of 2022. A community group ran an additional workshop and provided the researchers with those reflections.

#### 2.3.1 Recruitment to PPIE workshops

The Core PPIE Advisory Team encouraged us to seek diverse views, particularly from people who have experienced or are at higher risk of health inequalities. Therefore, we invited community groups and public contributors from a range of backgrounds, including diverse communities and under‐served areas, using known local and national networks. For example, the RDS, Fuse the Centre for Translational Research in Public Health, Coalition for Personalised Care and Community Catalysts advertised the event. We used numerous advertising methods including word of mouth, email and flyers. Expression of interest was through an online form, email or calling/texting the lead author. We also worked with several community groups with members from a wide range of socioeconomic backgrounds including representation from marginalised communities (including people with learning difficulties and experiencing ill‐health). As this was PPIE, involvement was open to anyone with no inclusion/exclusion criteria. All public contributors were informed about how the information would be captured, stored and used, including the use of direct but anonymised quotes in the outputs of the project, such as the animation, presentations, and publications. All public contributors provided informed consent either verbally or via email.

#### 2.3.2 Co‐production of PPIE activities and workshops

The workshops were co‐produced with members of the public through multiple meetings with the Core PPIE Advisory Team and one meeting with a community group. At these meetings, careful consideration was given to the aims, objectives and content of the public workshops (including the language), as discussing health inequalities can be challenging due to the stigma that can surround them.^14^ Changes were made iteratively such as adding a section to the second half of the workshops exploring possible solutions around reducing health inequalities to help the workshops end on a positive note. The evaluation form and debrief document (‘What Happens Next’) were also co‐produced with the Core PPIE Advisory Team.

We followed NIHR guidance on co‐production.^7^ Table 2 summarises how members of the public were involved in each stage of the project mapped onto the key principles of the NIHR co‐production guidance. In summary, everyone who was involved was of equal importance and all comments were carefully considered for inclusion. We endeavoured to help public contributors feel valued by listening and acting on comments and suggestions, thanking them for their time, feeding back changes that had been made (e.g., going back to the same community group with the next iterated version of the resource) and prompt remuneration in accordance with preferences. Being reliable, responsive to all communications and helpful (e.g., making adjustments to facilitate involvement where required and supporting public contributors to access vouchers when IT issues arose) were important to help build and maintain relationships.

**Table 2:** Summary of the project's PPIE involvement and impact mapped on to the NIHR co‐production key principles.^7^

Stages of the project	PPIE involvement and impact	Mapped on to the NIHR coproduction principles
Securing co‐leadership and funding	A member of the public was a co‐applicant on the funding bid. Co‐leads (researcher and member of the public) discussed and agreed how they would work together sharing responsibility and power at the start.	Sharing of power Reciprocity Everyone is of equal importance
Core PPIE Advisory Team Development	Co‐leads and UNFAIR PPIE members decided how they would work together including making decisions by consensus and adding regular updates about the progress of this project and the findings from the work packages from UNFAIR.	Sharing of power Building and maintaining relationships Everyone is of equal importance
Design of the workshops	The Core PPIE Advisory Team and a community group co‐produced the workshops' content including aims and objectives, the images to include and questions.	Including all perspectives Everyone is of equal importance
Advertising the workshops	The Core PPIE Advisory Team co‐produced the expression of interest form and flyer advertising the online workshop. Using national/local networks and a range of ways to advertise helped increase the diversity of public contributors. This included building new relationships and nurturing existing ones.	Including all perspectives and skills Building and maintaining relationships
Undertaking the workshops	The Core PPIE Advisory Team co‐chaired small groups or workshop discussions along with members of staff. Members of the public were actively involved in each workshop.	Including all perspectives and skills Everyone is of equal importance Building and maintaining relationships
Evaluating the workshops	The Core PPIE Advisory Team co‐produced the evaluation questionnaire and ‘What Happens Next’ document. Co‐leads reflected at all stages of the project capturing the learning.	Everyone is of equal importance
Final resource including script development	The Core PPIE Advisory Team was involved in the decision regarding the choice of the final resource (illustrations vs. animation). After feedback from both the Core PPIE Advisory Team and a community group, the following changes were made: Simplified and clarified the language. Rephrased the language to increase clarity, for example, advised to use active voice in the script.	Including all perspectives and skills Everyone is of equal importance Building and maintaining relationships
Animation development	Reviewed iteratively the storyboards and animation with the Core Advisory Group and a community group including increasing the diversity of characters, choice of buildings, adding more icons next to words, making font bigger and clearer as well as including subtitles. Members of the public participated in the voice‐overs for the animation.	Sharing of power Including all perspectives and skills Everyone is of equal importance Building and maintaining relationships
Dissemination	Animation is available to be used by anyone. All members of the public and community groups were involved in sharing the animation (e.g., on social media and requests to show it to other community groups).	Building and maintaining relationships Reciprocity

Abbreviations: NIHR, National Institute for Health and Care Research; PPIE, Patient and Public Involvement and Engagement; UNFAIR, UNderstanding Factors that explain Avoidable hospital admission Inequalities—Research study.

#### 2.3.3 Conduct of PPIE workshops

The online workshop was facilitated by a project researcher (J. L.) with small group discussions, of up to five public contributors, being facilitated by a member of the public from the Core PPIE Advisory Team and a researcher or a staff member who leads on PPIE in the organisation that they work for. The online workshops lasted 2 h. Two in‐person workshops were facilitated by the co‐leads (R. N. and C. P‐C.) and the third in‐person workshop was run entirely by members of the public. The in‐person workshops lasted for about 90 min.

**Figure 1:**
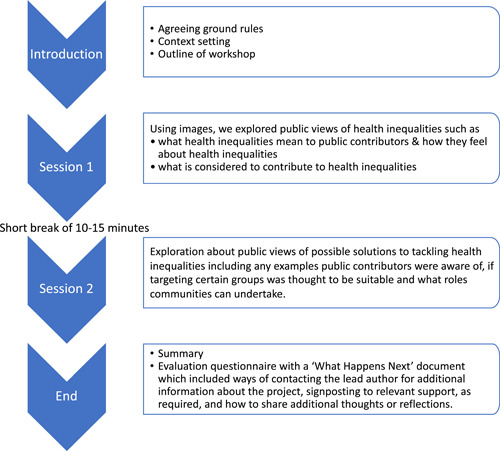
The planned structure for each workshop.

The planned structure for each workshop is detailed in Figure 1. Public contributors were shown two images that depicted health inequalities.^15^, ^16^ A table summarising healthy life expectancy^17^ and an image that outlined factors that relate to health^2^ were also included to facilitate a broader discussion upon recommendations made by our Core PPIE Advisory Team and a community group. The first half of the workshops explored how the public contributors viewed and felt about health inequalities. The second half was based on what solutions public contributors thought could reduce health inequalities.

In the online workshop to help generate discussion, a member of the public was invited to share their personal experiences of reducing hospital admissions and health inequalities.

To create a safe and supportive environment for public contributors, in addition to agreeing ground rules, a ‘What Happens Next’ document was shared with each public contributor. This two‐page document provided information about the next steps, ways to share additional thoughts (through email/phone call/text messages to the lead author or use of an online virtual board that allows adding posts onto a virtual board), as well as signposting to external support, if required, such as Citizens Bureau (an organisation that provides confidential advice on a range of subjects including debt and employment)^18^ and Samaritans (a charity that provides confidential emotional support).^19^ The document also contained contact details of the lead author to enable public contributors to get in touch as required. To capture feedback, a short questionnaire was also shared.

### 2.4 Resourcing of PPIE activities

The NIHR guidance for public contributors' remuneration was followed at each stage of the project.^20^ This was important to reduce barriers to involvement as it constitutes offering inclusive opportunities as part of the UK Standards for Public Involvement.^9^ To help fund the project, an application for a Tilly Hale award from Newcastle University was successful. This award alongside the NIHR UNFAIR funding (ref CA‐CL‐2018‐04‐ST2‐010) was used to fund our PPIE activities and the final resource (the animation). Remuneration of time was offered and was as flexible as possible including an offer of vouchers or monetary payments. Remuneration for involvement included involvement in the workshops, dissemination sessions and/or the voiceovers for the animation as well as reviewing documents, scripts and the animation as it developed. Public contributors were signposted to the Citizens Advice Bureau for advice, if required, about any potential implications to benefits from the remuneration.

### 2.5 Identifying the key messages from PPIE workshops

To aid recollection of what was said during the workshops, a mixture of notes and full transcription in verbatim from audio‐recordings were used depending on the workshop format. To support data management, the software system NVivo 1.6. was used.^21^ The transcriptions and notes were coded and analysed using inductive thematic analysis according to Braun and Clarke's framework.^22^ This approach is typically used in qualitative research but was applied to this work to strengthen how the key messages were identified.

### 2.6 Resource development

The Core PPIE Advisory Team with the research team considered two options for the resource, which were illustrations or an animation. The Core PPIE Advisory Team was split equally regarding which to choose; therefore, the vote was widened out to the research team. An animation to convey the key messages from the workshops had the most votes. A script was produced based on the workshops' key messages using verbatim extracts. The Core PPIE Advisory Team, UNFAIR research team members and a community group iteratively reviewed the script and animation ensuring the public's voice was integral to the animation.

### 2.7 Evaluation of the work

After each workshop and session, public contributors were asked to fill out a short questionnaire. Once the animation was launched, a reflective session was held with the Core PPIE Advisory Team.

## 3 KEY MESSAGES

A total of 58 people took part in the workshops. Most public contributors lived in the North East of England but public contributors from other parts of the country, including Yorkshire and Humber, North West England and West Midlands, also attended. There was a diversity of backgrounds including a range of ages and ethnicities, living in rural, urban as well as socioeconomically disadvantaged areas and people at risk of experiencing marginalisation (including people with learning disabilities, physical and mental health conditions). On many occasions, those taking part were experiencing overlapping and intersecting multiple layers of marginalisation and exclusion in their day‐to‐day lives.

### 3.1 Key messages from the workshops

The key messages are divided into two main themes:

1. the ways in which health inequalities are viewed by members of the public.
2. public views around how to address health inequalities.

#### 3.1.1 The ways in which health inequalities are viewed by members of the public

This theme was divided into four subthemes:

1. What health inequalities mean to members of the public.
2. The emotions behind health inequalities.
3. The factors that affect health and health inequalities across the life course.
4. The complexity of the system in which we live.

##### What health inequalities mean to members of the public

Health inequalities were considered as unfair differences in health between different population groups with their causes and consequences being complex. Health inequalities involved not only how long people live but also how good their health is. Essentially, some people were reported to be dying earlier than expected, as well as, having less healthy lives:

A lot of my peers didn't make it. Literally didn't make it. Aren't here anymore. (Online workshop)

##### The emotions behind health inequalities

Health inequalities conjured up powerful emotions such as anger, hopelessness, frustration, worry, annoyance and alarm.

It is shocking to see that there is an 18‐year difference [in healthy life expectancy] between the most and least deprived areas in England. (Online workshop)

“The health difference across the country matters …. What's on the map* is unfair. It shouldn't be as it is. The difference [in how long you live for] is down to where you live. (In‐person workshop) *The map from Bambra C and Orton C^22^ showed life expectancy for England at varying locations compared to the English average with areas being below, around or above the English average life expectancy

Not everyone was surprised about the differences in health, especially people who see these differences every day.

Public contributors were concerned and frustrated that the geographical health inequalities existed, that differences were present within and between areas. In particular, the North/South divide in health was discussed, which was felt to have become worse and greater since the COVID‐19 pandemic. Some public contributors were frustrated that these North/South health inequalities have ‘gone on for years’.

People were also concerned about the changes, particularly cuts to public spending, which was reported to have led to a worsening of the situation:

Anything that was there has been taken, [there is] nothing there and hard for deprived areas. (Online workshop)

All the public contributors that we spoke to wanted more work to be undertaken to tackle health inequalities and wanted to see meaningful change; that this was real life and not fiction. It was ‘high time’ that something was done to improve the situation.

##### The factors that affect health and health inequalities across the life course

These factors were considered complex and interconnected. This included the wider determinants of health, such as the wider socioeconomic and political system, access to good quality education, transport as well as healthcare and services, where people work and what they did for work, individuals' behaviours (which included both health‐harming behaviours, such as alcohol or substance misuse, and health‐promoting behaviours, such as healthy diet or exercise), how connected people felt, and particularly where people were born, lived and played:

Where you live is important, and you could be just a few miles from a better situation. (Online workshop)

Contact with family and friends, income, good housing, good community support. (In‐person workshop)

Also lifestyle (drinking, smoking, alcohol, exercise). If you have a lifestyle that keeps you healthy. (Online workshop)

Other factors included stress and how much money people had. For example, the cost of living was discussed and how this was affecting health, and forcing people to make difficult choices:

…a lot of people are forced between eating and getting heated. (In‐person workshop)

##### The complexity of the system in which we live

Concerns were raised that the system, which includes public services and benefits, is complex and becoming progressively hard to navigate.

You shouldn't have to learn a system in order to know how best to use it. (Online workshop)

Multiple barriers were discussed including services, support or care having gatekeepers, or having to jump through hoops. Some people also stated feeling degraded, judged and blamed while navigating the required processes. This was reported as being stressful and leading to less seeking of the necessary support.

…they've agreed now, he's eventually on the pathway for ADHD/possible autism, but it's been one door open, and then that door would just slam in our face. (In‐person workshop)

Also, there were concerns that people who are in power, which includes decision makers, are out of touch with how arduous life can be particularly in underserved communities:

I think they [decision makers] live in a different world. (In‐person workshop)

#### 3.1.2 Public views on how to address health inequalities

This theme was divided into three subthemes:

1. Recognising the importance of communities and individuals.
2. Ways to help people have better health.
3. Partnership working.

##### Recognising the importance of communities and individuals

It was reported that respecting, valuing and accepting everyone was vital. People wanted to feel heard, not dismissed and their lived experience considered.

All patients are people with feelings; they aren't numbers, they're not statistics. (Online workshop)

Like, we might be in an underprivileged area to where people look, but we're not. Like, we're just us and we're getting by the best we can. (In‐person workshop)

It was highlighted that decision makers needed to recognise the strength of individuals and communities. Listening to, working with, and empowering individuals and communities to design what works for them from start to finish is a way to do this; communities and individuals should be front and centre of the decision making.

It starts with a conversation and listening, not having ideas ready. (Online workshop)

Local communities know what local communities need. (Online workshop)

##### Ways to help people have better health

There was a need to consider not only treatment but prevention of health problems, which included acknowledgement that public health and public health policy was part of this and not just down to individuals.

[Think] Bigger picture—spend 100s of billions of pounds treating people in hospital, but shouldn't we be looking upstream, and a little bit more investment in public health and health prevention, I think, is the way forward. We spend a lot on treating. But we need to spend on prevention. Local authorities having monies on public health budgets being cut back. We need to stop people becoming unhealthier. (Online workshop)

Providing education and opportunities as well as developing a sense of belonging and community were important. The message needed to be easy to understand and trusted. Getting the right message delivered by the right person to the right people in the right way was highlighted as crucial:

Working with trusted people, citizens, business, communities from all age groups to help deliver key healthcare messages. (Online workshop)

It was reported that services and support should be welcoming, accessible and simple to use by everyone. This included services and support being easy to understand and culturally appropriate as well as providing consistency of care. For example, tailoring the support to that particular person to meet their needs and giving choices about the provision of support or care are important.

Should be services centred around the person not the postcode. (Online workshop)

We're not numbers: one size does not fit all. (Online workshop)

Some groups of people may benefit from extra support:

It makes sense to target different groups because they want and need different things. (Online workshop)

However, care was required over labelling people or groups, such as ‘rich’ versus ‘poor’ or ‘deprived’ versus ‘not deprived’ areas, as labels can trigger negative emotions as well as create barriers:

When we [are] saying ‘can you kind of target particular groups that you know’ […], maybe you need to but also if you put a label on something then people won't go, so I think that's about things being stigmatised. (Online workshop)

##### Partnership working

Public contributors reported several stakeholders were required to help reduce health inequalities, such as central and local government, the media, healthcare professionals, community groups, researchers, communities and individuals. By breaking the problem down and problem solving together, meaningful change around reducing health inequalities was felt to be achievable.

… that's the approach for me that really works, bringing together people from all parts of this discussion in one place tackling one subject. (Online workshop)

### 3.2 Impact of PPIE on the resource development and project

Members of the public were an integral part of the project and resource development. Table 2 provides a summary of the ways the PPIE influenced the project and final resource (animation). The animation provides a condensed version of the key messages and is found at bit.ly/animationUNFAIR. The animation is freely and publicly available for anyone, including decision makers, to use. The animation has been shared with public contributors who attended the online workshop via email and with each of the community groups that were involved through an in‐person dissemination session.

### 3.3 Evaluation (workshops and animation)

During the workshops and dissemination sessions, we had 80 contacts with public contributors outside the Core PPIE Advisory Team (as a person could be involved more than once) of which 58.8% completed the evaluation form. A total of 93.6% found the information about the workshop to be very easy or easy to understand with the remainder reported being adequate or was left blank. A total of 91.5% stated they felt their input had been valued with the remainder reported sometimes felt valued, did not know if their input was valued or had been left blank. We have been monitoring social media activity since the launch including number of views of the animation. The animation has been widely promoted, for example, through the NIHR, NHS England and the Association of Directors of Public Health's network.

## 4 DISCUSSION

While overlap regarding the use of similar methods can occur with PPIE and qualitative research, it is the intention of the project that distinguishes the two.^23^ This project was a PPIE project, not research, as the intent was to involve members of the public to produce a resource that addressed uncertainties centred around what the term ‘health inequalities’ means to people rather than generate new knowledge or research data.^23^ The project was in response to our Core PPIE Advisory Team identifying the need to be able to communicate complex concepts, such as health inequalities, when involving and engaging with members of the public about related research or work. Our Core PPIE Advisory Team encouraged the research team to seek diverse views to produce a resource to this end to share public views of health inequalities. Therefore, the project's purpose was to collaborate with a diverse group of people using co‐leadership and co‐production to create this resource. We did this by weaving collaborative design in multiple ways underpinned by the NIHR definition of public involvement and co‐production. This project's co‐production involved all three types of co‐production as identified by Voorberg et al.,^24^ which included initiating the project (initiators) as well as members of the public designing (co‐designing) and delivering the project including the workshops (co‐implementers) with researchers. For example, involvement started early with a successful application for a Tilly Hale award and one of the co‐leads was a member of the public as well as one of our Core PPIE Advisory Team. This award enabled the early involvement of public contributors in the co‐design of the workshops. The co‐leads (a member of the public and a researcher) worked closely together reviewing key decisions including when and how to involve either the Core PPIE Advisory Team or the wider network of public contributors. The content and running of the workshops were co‐produced with the public, including regular meetings with the Core PPIE Advisory Team sharing invaluable feedback and ideas. Inclusive involvement opportunities were offered with flexible ways of involvement and remuneration.

The animation, based entirely upon the key messages from the workshops, was co‐produced in partnership with members of the public and a local film‐making company ensuring the public's voices represented a golden thread throughout the final resource. This was enabled by the steps taken at every stage of the project to be as inclusive as possible and by encouraging active involvement to embrace co‐production and meaningful PPIE. The approach was innovative as members of the public were involved in an element before the research process starts that they are not typically involved with which was the understanding of the public health concept (health inequalities) that the research study UNFAIR is investigating. Communicating complex concepts like health inequalities clearly is the first step to increasing the involvement of members of the public as public contributors or research participants in this specific research project or related research topics.

From the research‐base, lay perceptions of health and health inequalities have been explored in the United Kingdom with most exploring adults' views, including socioeconomically advantaged and disadvantaged areas^25^, ^26^, ^27^, ^28^, ^29^, ^30^ to marginalised groups such as the Traveller community.^31^ A subsection of the literature exploring children's and young people's views exists.^32^, ^33^, ^34^, ^35^, ^36^ However, the research findings around public understanding of health inequalities can be conflicting, likely because of the varying methods used and the way the questions were framed. When the body of the literature is considered, lay perceptions recognise the complexity of several contributing factors resulting in health inequalities including macrostructural, place‐based, psychosocial, material and individual's lifestyle/behavioural components.^5^, ^14^, ^25^, ^26^, ^30^, ^31^, ^36^ Furthermore, discussing health inequalities can be emotive, for example, emotions reported in the literature include helplessness,^31^ worry,^25^ inferiority, shame and concern about being judged particularly when comparison between social groups occurred^30^ as well as feeling stigmatised.^25^, ^33^, ^36^ In particular, shame has been reported due to concerns of ‘being “looked down on”’^36^ ^(p. 9)^ and how other people may have wrong perceptions regarding the reasons for any money difficulties, for example being falsely perceived as lazy, or the shame around ‘living in the “wrong” kind of housing’.^33^ ^(p. 9)^ However, despite the negative perceptions and treatment by others, not everyone felt shame; some felt anger and resentment.^29^

Concerns about being judged can result in a reluctance to seek support.^25^ Labelling was often rejected, especially if contained assumptions about the way in which people lived their lives and what could then be inferred about them.^14^, ^26^ This rejection is not indicative of all groups as some groups are reported to have less issues with labelling their health inequalities, for example, the Traveller community as described by Hodgins et al.^31^ This was hypothesised to be due to the already politicised context and the type of identity (social vs. ethnicity).^31^ These are some examples and do not encompass the views of other marginalised communities or consider intersectionality.

In our workshops, we also found that discussing health inequalities can evoke strong emotions. Additionally, the importance of being valued, respected and listened to, as well as not being judged, were highlighted. The concept of targeting people according to need was considered potentially worthwhile. However, there was a potential cost of labelling people and fuelling the associated stigma. From our PPIE workshops, we found that care is required around the language used when considering targeting certain groups to reduce health inequalities; it may be beneficial to seek the views of the targeted groups to get this right.

### 4.1 Reflections and learning

The key learning is summarised in Table 3. This type of work involves a substantial amount of commitment, time and resource, which should not be under‐estimated. However, it was viewed that the amount of commitment and effort that is put into these types of projects pays in dividends regarding the strength of relationships that are built as well as the final product. Also, challenges are inevitable, for example, some suggestions or feedback may conflict with others' points of view. There were occasions when it was not possible to act on all the feedback due to available time, resource or it was at odds with other feedback. Therefore, having an approach to resolve these types of challenges is important. Some of the approaches we used included a voting system, sense‐checking decisions and rationale behind the decisions with the wider team or seeking to convey the views of the majority.

**Table 3:** Summary of the key learning from this PPIE project.

Key learning	Explanation
Ways of working	Consider how members of the public will be involved at each stage of the project in accordance with budgetary constraints.
	When working closely with a defined group of PPIE contributors and/or PPIE coapplicant, decide together from the offset about how best to work on the project. Tools exist to assist with this planning.^7^, ^11^, ^12^, ^13^
	Decide in advance how to deal with any challenges as a team, including potentially dominant contributors, which may occur in focus groups/workshops.
	Two facilitators per group work well.
Diversity and inclusion	Remove barriers to involvement as much as possible including providing options for involvement.
	Language matters, keep it simple and clear with review by PPIE contributors.
	Link in with networks/organisations who work with the audience of interest to promote the workshops/opportunities especially when not a defined ‘group’.
Time	This type of work takes time to build relationships, so it is vital to keep people informed and adjust according to needs where possible.
	Understand the process for remuneration in the relevant organisations before any workshops to reduce payment delays.
	Always include additional project time for unexpected eventualities that invariably occur.
Challenges	Any challenges that occur with co‐production and PPIE are opportunities for growth.
	Some feedback may conflict so worth deciding how to approach this when this occurs (e.g., use of the voting system).

Abbreviation: PPIE, Patient and Public Involvement and Engagement.

### 4.2 Strengths and limitations

We used a collaborative and inclusive approach at all stages of the project to ensure that members of the public were actively involved. We considered the potential sensitivities around the topic area and had structures in place to support public contributors, if required. We received consistently positive feedback that most public contributors felt their views were valued and that the information was easy or very easy to understand. Being explicit in every workshop about how public contributors' views were valued appeared to be important on top of the existing positive body language and words of appreciation. Therefore, this feedback has informed our practice moving forward. A robust analytical approach underpinned by a theoretical framework, typically used in qualitative research methods, was used to strengthen and increase the key messages' rigour.

Conducting one of the workshops online may have hindered some public contributors from being able to contribute; therefore, in‐person workshops were offered to help overcome this barrier. This was a public involvement project and not research. The key messages are not necessarily representative of the whole population but summarises the key messages from the people we spoke to. It is also worth being mindful that even when members of the public share a similar factor, such as living in the same area, a diversity of views is likely and homogeneity should not be assumed. Whilst we aimed to seek as diverse views as possible given the limited time and resources available, it was not possible to reach all groups, for example, children. Additionally, due to these limitations, we were not able to involve every public contributor at each stage.

### 4.3 Implications for practice/policy and further research/PPIE

There are multiple practice and policy impacts of this work. For example, health inequalities are currently a major focus of research both locally and nationally, but there are limited accessible resources around how the public view and feel about health inequalities. This project, therefore, fills this gap. The animation, designed with longevity in mind, can be used as the building blocks for engagement with the public and can help fulfil the levelling‐up recommendation of having a conversation with the public about levelling up health.^6^ The animation will enable researchers to progress conversations around health inequalities as it acts as a resource to communicate the concept of health inequalities to the public. The co‐produced animation highlights real‐life experiences of health inequalities and provides a way for the public's voice to have an impact on policy and practice. It does this by providing examples of what health inequalities mean to people and corroborates the moral argument for decision makers to act. There is a lack of examples of PPIE involving members of the public in the exploration of more complex/abstract concepts, such as health inequalities and public health, as PPIE often involves a specific patient group with a particular condition. This project is important as it also bridges this gap and provides an example for other researchers looking to undertake related projects.

## 5 CONCLUSION

This project is an example of fully engaged and inclusive PPIE achieved through co‐leadership and co‐production. The animation provides a route for the public's voice to influence policy and practice by informing the understanding and action to address health and care inequalities. The animation provides a description of what health inequalities means to people with examples of lived experience and substantiates the moral argument for action by decision makers. The project also provides an example of an inclusive PPIE journey for other researchers considering PPIE around complex concepts for public health research.

## AUTHOR CONTRIBUTIONS

Charlotte Parbery‐Clark co‐led the design and facilitation of all the workshops (apart from the one that was led by a community group); thematic analysis of transcripts/facilitators' notes; wrote the manuscript. Rosemary Nicholls co‐led the design and facilitation of all the workshops (apart from the one that was led by a community group); co‐led the development of the script and animation; reviewed and edited the manuscript. Lorraine McSweeney assisted with the delivery of the online workshop; supported the interpretation of the thematic analysis; assisted with the development of the script and animation; reviewed and edited the manuscript. Sarah Sowden is the principal investigator for the UNFAIR research project, assisted with the design of the workshops and delivery of the online workshop; supported the interpretation of the thematic analysis; assisted with the development of the script and animation; reviewed and edited the manuscript. Joanne Lally assisted with the design of the workshops, delivery of the online workshop and UNFAIR PPIE Core Advisory Team dissemination session; supported the interpretation of the thematic analysis; assisted with the development of the script and animation; reviewed and edited the manuscript. All authors read and approved the final manuscript.

## CONFLICT OF INTEREST STATEMENT

The authors declare no conflict of interest.

## Supporting information

Supporting information.

## Data Availability

The data that support the findings of this study are available from the corresponding author upon reasonable request.
